# Quaternary ammonium iminofullerenes improve root growth of oxidative-stress maize through ASA-GSH cycle modulating redox homeostasis of roots and ROS-mediated root-hair elongation

**DOI:** 10.1186/s12951-021-01222-7

**Published:** 2022-01-04

**Authors:** Fuju Tai, Shuai Wang, Benshuai Liang, Yue Li, Jiakai Wu, Chenjie Fan, Xiuli Hu, Hezhong Wang, Rui He, Wei Wang

**Affiliations:** 1 National Key Laboratory of Wheat and Maize Crop Science, College of Life Science, Henan Agricultural University, Zhengzhou, 450002 China; 2 NanoAgro Center, College of Plant Protection, Henan Agricultural University, Zhengzhou, 450002 China

**Keywords:** Iminofullerene, Hydrogen peroxide, Root hair, ROS, ASA-GSH cycle, NADPH oxidase

## Abstract

**Background:**

Various environmental factors are capable of oxidative stress to result in limiting plant development and agricultural production. Fullerene-based carbon nanomaterials can enable radical scavenging and positively regulate plant growth. Even so, to date, our knowledge about the mechanism of fullerene-based carbon nanomaterials on plant growth and response to oxidative stress is still unclear.

**Results:**

20 or 50 mg/L quaternary ammonium iminofullerenes (IFQA) rescued the reduction in root lengths and root-hair densities and lengths of *Arabidopsis* and maize induced by accumulation of endogenous hydrogen peroxide (H_2_O_2_) under 3-amino-1,2,4-triazole or exogenous H_2_O_2_ treatment, as well as the root active absorption area and root activity under exogenous H_2_O_2_ treatment. Meanwhile, the downregulated contents of ascorbate acid (ASA) and glutathione (GSH) and the upregulated contents of dehydroascorbic acid (DHA), oxidized glutathione (GSSG), malondialdehyde (MDA), and H_2_O_2_ indicated that the exogenous H_2_O_2_ treatment induced oxidative stress of maize. Nonetheless, application of IFQA can increase the ratios of ASA/DHA and GSH/GSSG, as well as the activities of glutathione reductase, and ascorbate peroxidase, and decrease the contents of H_2_O_2_ and MDA. Moreover, the root lengths were inhibited by buthionine sulfoximine, a specific inhibitor of GSH biosynthesis, and subsequently rescued after addition of IFQA. The results suggested that IFQA could alleviate exogenous-H_2_O_2_-induced oxidative stress on maize by regulating the ASA-GSH cycle. Furthermore, IFQA reduced the excess accumulation of ROS in root hairs, as well as the NADPH oxidase activity under H_2_O_2_ treatment. The transcript levels of genes affecting ROS-mediated root-hair development, such as *RBOH B*, *RBOH C, PFT1*, and *PRX59*, were significantly induced by H_2_O_2_ treatment and then decreased after addition of IFQA.

**Conclusion:**

The positive effect of fullerene-based carbon nanomaterials on maize-root-hair growth under the induced oxidative stress was discovered. Application IFQA can ameliorate oxidative stress to promote maize-root growth through decreasing NADPH-oxidase activity, improving the scavenging of ROS by ASA-GSH cycle, and regulating the expressions of genes affecting maize-root-hair development. It will enrich more understanding the actual mechanism of fullerene-based nanoelicitors responsible for plant growth promotion and protection from oxidative stress.

**Graphical Abstract:**

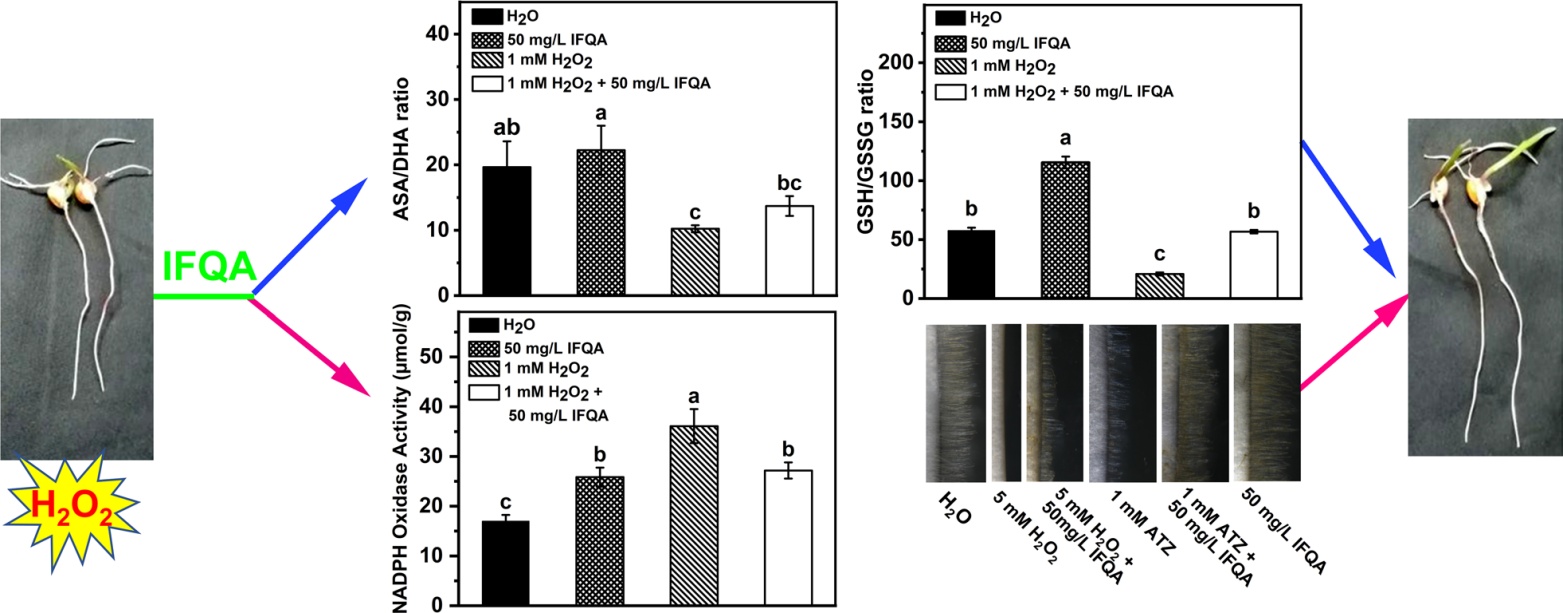

## Introduction

In plants, reactive oxygen species (ROS) are proved to be involved in various processes of plant growth and development, as shown for seed germination [1–3], leaf development [4], pollen tube growth [5, 6], and root hair development [6–11], as well as plant defense from environment stresses, such as drought, salinity, and heavy metal [12–15]. Among all ROS, including hydrogen peroxide (H_2_O_2_), superoxide radical and hydroxyl radical, H_2_O_2_ is often proposed as the most important signaling molecule because of its long lifespan and diffusibility [16, 17].

There is evidence that the function of H_2_O_2_ in plants is concentration-dependent [18]. H_2_O_2_ at a lower concentration (< 100 μM) promoted cell expansion and an increase in root diameter, conversely, 100–500 μM H_2_O_2_ inhibited root elongation of rice [18]. An increased number of studies indicated that exogenously-sourced H_2_O_2_ at a lower level acts as an important signaling molecule in the development of plants and stress response [19–22]. At elevated levels, it triggered oxidative burst to result in oxidative damage of cell membranes, proteins, DNA and RNA, and even destruction of cells and death of the organism [23, 24].

In plant roots, NADPH oxidases (respiratory burst oxidase homologs, RBOHs) is one of the sources for H_2_O_2_ production, which is excess accumulated due to extreme environmental stresses (i.e., drought, saline, and high light) resulting in the oxidative stress [25–27]. NADPH oxidases play crucial roles in plants response to stress and also participate in the developmental processes of roots and root hairs [25–27]. To prevent ROS from reaching damage levels, some small antioxidant molecules, including glutathione (GSH) and Ascorbic acid (ASA), and antioxidant enzymes, including glutathione reductase (GR), monodehydroascorbate reductase (MDHAR), dehydroascorbate reductase (DHAR), and ascorbate peroxidase (APX), in ASA-GSH cycle, are vital to maintain an appropriate ROS levels and cell redox balance [28–31].

More and more studies indicated that fullerene-based carbon nanomaterials, particularly water-soluble derivatives of fullerenes, have positive effects on plant growth under various stresses. Upon fullerenols C_60_(OH)_27_ treatment, seed germination, biomass accumulation, and antioxidant system in *Brassica napus L.* was upregulated under water stress [32]. In another study, salt tolerance and phosphorus uptake of wheat seeds were enhanced through increasing of H_2_O_2_ neutralizing enzymes when seeds were pretreated with fullerenols C_60_(OH)_20_ [33]. Oxidative damage caused by drought stress was alleviated in sugar beets by fullerenol nanoparticles foliar application [34]. Polyhydroxy fullerene C_60_(OH)_24_ could prevent oxidative stress caused by UV-B radiation, salt stress, and the excess of salicylic acid, and promote root growth [35]. A similar phenomenon was observed in our previous study that seed germination of maize under polyethylene glycol (PEG) stress was promoted by fullerenol nanoparticles [C_60_(OH)_22_·8H_2_O]_n_ [36]. Recently, our group reported cationic and water-soluble fullerene-based nanoparticles, quaternary ammonium iminofullerenes (IFQA), which can improve seed germination of maize and *Arabidopsis* by accelerating storage proteins degradation [37], and enhance maize-root elongation under PEG-stress conditions by improving the antioxidant system and expression of stress-related proteins [38]. The results indicated that IFQA can act as a nanoregulator to enhance plant seedlings responses to osmotic stress. However, it is still unclear whether the IFQA-mediated positive effect takes place under conditions leading to high oxidative stress.

Herein, the present study was the continuation of an investigation on plant root growth to reveal the effect of IFQA (what and how) during oxidative damage under different manipulation of H_2_O_2_ levels. The phenotypic analysis was carried out to assess the promotion effects of IFQA on root and root-hair growth of *Arabidopsis* plantlets and maize seedlings under oxidative stresses. The physiological assay was performed, including diaminobenzidine (DAB) staining to explore the effect of IFQA on H_2_O_2_ accumulation in maize-root tips, the physiological indexes of GSH-ASA cycle in maize roots to analyze the effect of IFQA on cell antioxidant potential, and NADPH oxidase activity. Furthermore, the IFQA-mediated expressions of genes affecting ROS-mediated root hair development was also investigated.

## Materials and methods

### Size distribution and zeta potential assay

The preparation method of IFQA has been published by our group; the molecular formula is C_60_(NCH_2_CH_2_NH_3_^+^CF_3_COO^−^)_4_·10H_2_O [37]. The measurement of the zeta potential, average hydrodynamic diameter (HD), particle size distribution, and polydispersity index (PDI) through dynamic light scattering (DLS) using a Nanotrac Wave II particle size&zeta potential analyzer (Microtrac Inc., USA).

### Plant materials and treatments

Maize seeds (*Zea mays* L. Zhengdan 958) were thoroughly washed and soaked in water for 24 h for imbibitions, placed in culture dishes with water, and incubated at 25 °C for germination. After 2 days, the seedlings with root consistent growth were transferred on filter paper with H_2_O, 50 mg/L IFQA, 1 mM H_2_O_2_, 1 mM H_2_O_2_ + 50 mg/L IFQA, 5 mM H_2_O_2_, 5 mM H_2_O_2_ + 50 mg/L IFQA, 1 mM 3-amino-1,2,4-triazole (ATZ, catalase inhibitor), 1 mM ATZ + 50 mg/L IFQA, 3 mM ATZ, and 3 mM ATZ + 50 mg/L IFQA culture dishes, planted in a growth chamber with a relative humidity of 75% and 16/8 h day/night cycle at 25 °C, and cultured for 3 days, respectively. The seedlings were phenotypic analyzed and stained by DAB, and the root tips were collected to measure physical factors and to extract RNA for analysis of gene expressions.

*Arabidopsis* (*A. thaliana*, Col-0) seeds were surface sterilized and planted on Murashige and Skoog (MS), MS + 0.3 mM H_2_O_2_, MS + 0.3 mM H_2_O_2_ + 20 mg/L IFQA, MS + 20 mg/L IFQA, MS + 2 µM ATZ, MS + 2 µM ATZ + 20 mg/L IFQA, MS + 3 µM ATZ, MS + 3 µM ATZ + 20 mg/L IFQA, MS + 1 mM buthionine sulfoximine (BSO, an inhibitor of GSH synthesis), and MS + 1 mM BSO + 20 mg/L IFQA. The plantlets were vertically grown in a growth chamber under a 16/8 h day/night cycle at 22 °C. At 7 days after planting, the seedlings were phenotypic analyzed and stained by 10-acetyl-3,7-dihydroxyphenoxazine (ADHP).

### Phenotypic analysis of roots and root hairs

The maize-root hairs were photographed at 8–12 h after transferring. The root lengths were measured at 72 h after the transferring. The mean value was obtained by statistics 30 roots for each replicate.

The roots of 7-d-seedling *Arabidopsis* were photographed, and the root lengths were calculated by measuring 30 roots for each replicate. For analysis of root hair, the area about 10 mm away from the root tip of 7-d-seedling *Arabidopsis* as root-hair zone was photographed, and the root hairs under the field of view of the lens were measured by using Image J software. The root-hair number of at least 30 independent *Arabidopsis* plantlets and the lengths of more than 600 root hairs were measured for each treatment.

### Measurements of root active absorption area and root activity

Maize roots were stained by methylene blue according to the described method to reveal root active absorption area (RAA), which was closely related to the ability of roots to absorb water and nutrients [38]. 2,3,5-triphenyltetrazolium chloride (TTC) staining assay was common used to evaluate root activity through measurement of respiratory activity. The colorless TTC can be reduced by living tissues to the red triphenyl formazan as a result of the dehydrogenase activity of the mitochondrial respiratory chain. Maize-root tips were cut off about 1.5 cm and soaked in the staining solution (1% TTC with phosphate buffer solution at pH 7.5) to avoid light. After shaking for 20 min, the stained root tips were photographed under anatomical lens (Olympus, Japan).

### Contents of ASA, GSH, dehydroascorbic acid, and oxidized glutathione

The contents of ASA, GSH, dehydroascorbic acid (DHA), and oxidized glutathione (GSSG) in maize roots were measured according to the corresponding kits (Solarbio, China) as described in the previous research [38].

### Activities of antioxidant enzymes and contents of H_2_O_2_ and malondialdehyde

Briefly, maize root tips (0.2 g) were homogenized in a mortar with 0.2 ml phosphate buffered saline on ice. The homogenate was transferred into a centrifuge tube and centrifuged for 20 min with 12,000*g* at 4 ℃. The supernatant was used to measure the activities of MDHAR, DHAR, GR, and APX according to the manufacturer’s instructions of kit (Solarbio, China). The contents of H_2_O_2_ and malondialdehyde (MDA) in maize roots were also measured according to the corresponding kits (Solarbio, China) as described in the previous research [38].

### DAB staining

To assess the level of H_2_O_2_ accumulation in tissue by DAB staining, maize roots were stained by DAB dye solution (10 mM Na_2_HPO_4_ containing 1 mg/mL DAB) according to the previous research [38].

### ADHP staining

ADHP (0.025 g), a kind of H_2_O_2_ fluorescent probe, was dissolved in 1 ml dimethyl sulphoxide, and diluted with phosphate buffer solution seven times to stain *Arabidopsis* roots for 1 min. The root-hair zones of the stained roots were photographed by confocal microscope (Nikon, Japan).

### RNA extraction and real-time fluorescence quantitative PCR

Total RNA was isolated from the 5-d-maize roots using Trizol RNA extraction method according to the manufacturer’s instructions. The concentration and quality were measured by a NanoDrop ND-2000 (NanoDrop Technologies, Wilmington, DE, USA). After removing contaminative genomic DNA, cDNA was synthesized using reverse transcription kit, and real-time fluorescence quantification was performed using SYBR Green (Shanghai, China) in an ABI Stepone Plus real-time PCR system with UBI as internal control. The primers of genes were designed using Premier 5 software (Premier Biosoft, Palo Alto, CA, USA) and synthesized by Sangon (China). A list of real-time fluorescence quantitative PCR (qRT-PCR) primers is provided in Table 1. The amplification program was as follows: 10 min at 95 ℃, 40 cycles of 15 s at 95 ℃, 10 s at 60 ℃. Relative gene expression was evaluated using the 2^−△△Ct^ method. Each treatment had three replicates.

**Table 1:** Information of gene locus identifiers (IDs) and primer sequences used for qRT-PCR analysis

Gene name	Gene ID	Primers
*RBOH B*	100037794	L: 5’-GCCAAGCACTAAGTCAGAACCTAGC-3’ R: 5’-TGAACAGTCCAGCCATTATTCCAATCC-3’
*RBOH C*	100101532	L: 5’-CCTGAAGGGCTTGGCTACATTGAG-3’ R: 5’-TCTGGCTTAGTGCTTGGCTTGTG-3’
*RBOH H*	103635232	L: 5’-CTGAAGGAGTTTTGGGAGGAGATGAC-3’ R: 5’-CCGAGGCACTTAGCACGATGAC-3’
*RBOH J*	103650368	L: 5’-TGTGACAAGAACGGTGATGGTAAGC-3’ R: 5’-CGCAGCGTGTTTCTTCAGTTTAGC-3’
*PFT1*	821061	L: 5’-TCTACTTGTGAAGGTCTTGCTGAAGC-3’ R: 5’-CAGGTGTTGGCAGAGGATAAGGATTAC-3’
*PRX59*	100272764	L: 5’-CAACGCCTACTACAAGAACCTCCTG-3’ R: 5’-CAGAAGAAGTGCTCGCTGTCCTG-3’
*ZmSCR*	100382261	L: 5’-TCCGCCTCCTCTCACTCCTTATTG-3’ R: 5’-GCTTCTTGGTGGTCTAGCTGATGG-3’
*RHD6*	842965	L: 5’-CACACACTCCTCCAGAACGAACAC-3’ R: 5’-TGCTGGTTACTTAGTTGTAGACGAAGG-3’
*UBI*	103626648	L: 5’-GGAGTCTTCGGATACCAT-3’ R: 5’-CATGCCAGTCAATGTCTT-3’

### Statistical analysis

Statistical calculations were performed by DPS 8.0 software package. The results were displayed as mean ± standard error (SE). Least significant difference test was used to determine significant differences among treatments. Differences at P < 0.05 were considered significant.

## Results

### Size distribution and zeta potential of aqueous IFQA solution

**Fig. 1:**
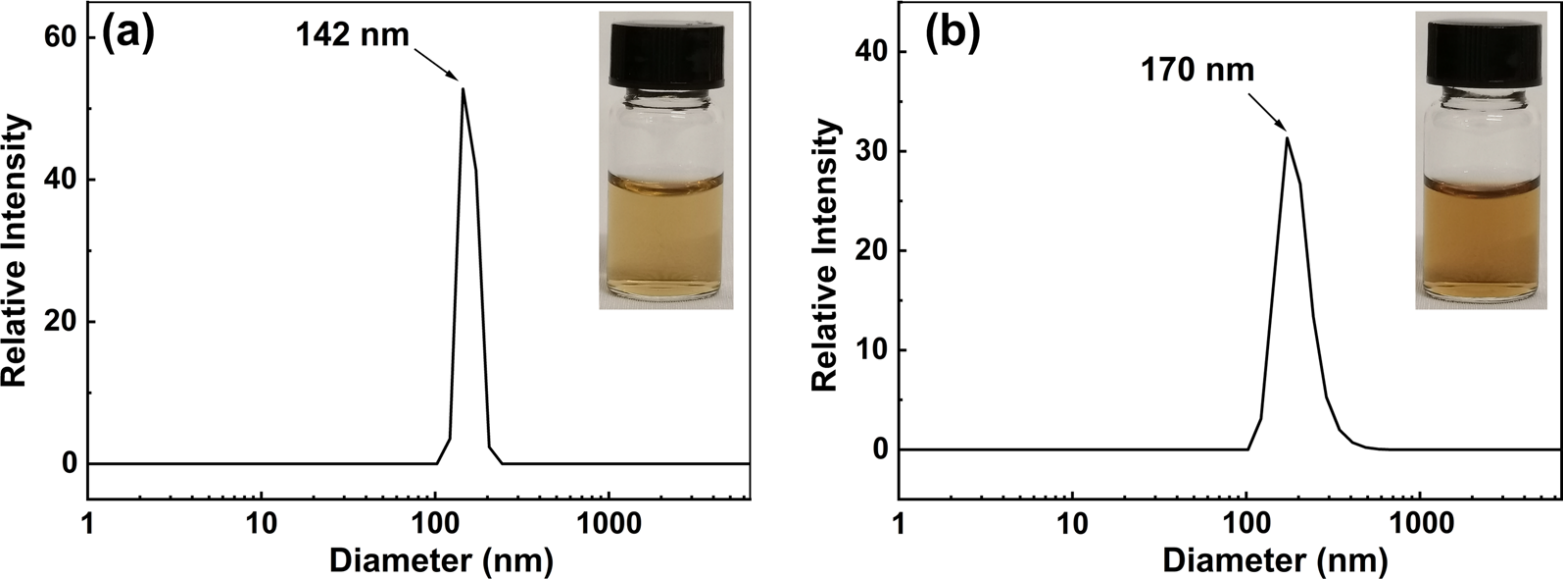
Size distribution of nanoparticles in 20 (**a**) and 50 (**b**) mg/L IFQA solution obtained by DLS. The insets display the colour of 20 (**a**) and 50 (**b**) mg/L IFQA solution

As shown in Fig. 1, 20 and 50 mg/L IFQA in deionized water were brownish yellow and had a similar tendency to aggregate. Figure 1a and Table 2 exhibit a monomodal nano-sized distribution from 120 to 200 nm for 20 mg/L IFQA solution, and the mean HD and PDI were 142 nm and 0.012, respectively. 50 mg/L IFQA solution had a slight increase in the size distribution from 120 to 500 nm, and the mean HD and PDI were 170 nm and 0.088 (Fig. 1b and Table 2). Unexpectedly, the zeta potentials of nanoparticles in the 20 and 50 mg/L IFQA solutions were >  + 200 mV, which exceeded the limits of the particle analyzer (Table 2).

**Table 2:** Mean size, size distribution, PDI, and zeta potential of IFQA aqueous solution

IFQA (μg/mL)	Mean size (nm)	Size distribution (nm)	PDI	Zeta potential (mV)
20	142	120–200	0.012	> + 200
50	170	120–500	0.088	> + 200

### Root growth of ATZ- or H_2_O_2_-stress maize and ***Arabidopsis***

**Fig. 2:**
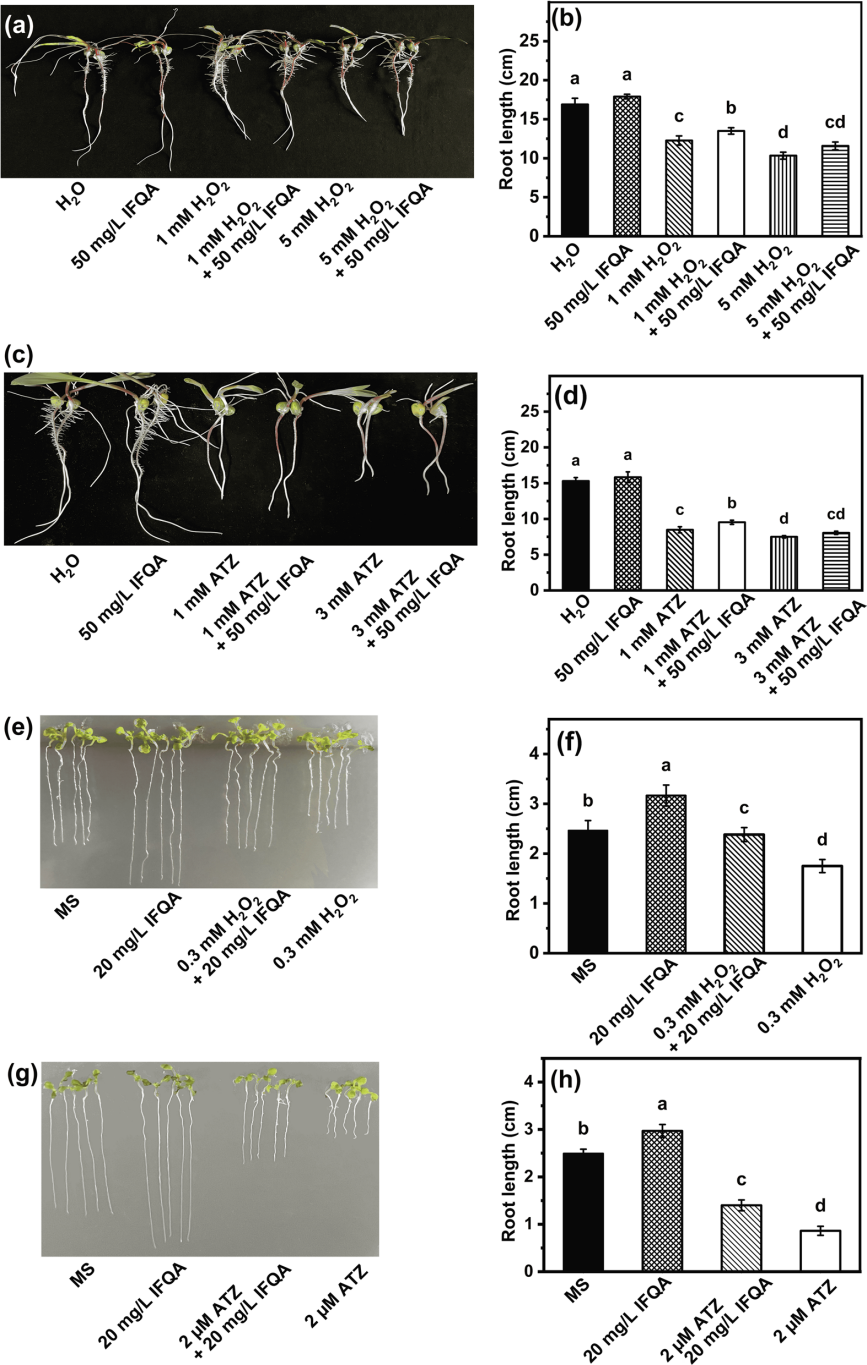
The effects of IFQA on the maize- and *Arabidopsis*-root lengths under H_2_O_2_ and ATZ treatment. The phenotypes (**a** and **c**) and root lengths (**b** and **d**) of 5-d-old maize seedlings, which were cultured in water at two days after germination and then transferred into water, 50 mg/L IFQA, 1 mM H_2_O_2,_ 1 mM H_2_O_2_ + 50 mg/L IFQA, 5 mM H_2_O_2_, 5 mM H_2_O_2_ + 50 mg/L IFQA, 1 mM ATZ, and 1 mM ATZ + 50 mg/L IFQA for 3 days; The phenotypes (**e** and **g**) and root lengths (**f** and **h**) of *Arabidopsis* plantlets, which were planted on MS, MS + 20 mg/L IFQA, MS + 0.3 mM H_2_O_2_ + 20 mg/L IFQA, MS + 0.3 mM H_2_O_2_, MS + 2 µM ATZ, and MS + 2 µM ATZ + 20 mg/L IFQA for eight days. Letters above the boxes indicate statistically significant differences between samples (P < 0.05)

Figure 2a presents the appearance of representative maize seedlings under various treatments. The root lengths in the 1 mM and 5 mM H_2_O_2_ treatment groups are reduced relative to that of the control (Fig. 2b): the mean lengths of the 1 mM (12.27 ± 0.59 cm) and 5 mM H_2_O_2_ (10.32 ± 0.45 cm) treatment groups are reduced by 27.4% and 39.9% relative to the control group (16.89 ± 0.80 cm), respectively; the inhibition by H_2_O_2_ was partially rescued by application of IFQA. The mean value (13.5 ± 0.41 cm) of root length in the IFQA + 1 mM H_2_O_2_ treatment group was significantly increased by 10.6% compared to that of the 1 mM H_2_O_2_ treatment group; 11.58 ± 0.50 cm of the IFQA + 5 mM H_2_O_2_ treatment group relative to that of the 5 mM H_2_O_2_ treatment group was more significantly increased by 12.2%. In addition to the restorative effect of IFQA on plant root growth under exogenous H_2_O_2_ treatment, it also has a similar function for regulating root growth under ATZ treatment caused endogenous H_2_O_2_ accumulation (Fig. 2c). As shown in Fig. 2d, the inhibition of 1 mM ATZ on maize-root growth (8.49 ± 0.42 cm) was partially restored to 9.54 ± 0.28 cm by IFQA, however, the recovery under 3 mM ATZ treatment was not obvious. Furthermore, the roots under IFQA treatment alone exhibited the longest length among all the treatment groups (Fig. 2a–d).

A similar phenomenon was observed in *Arabidopsis* plantlets (Fig. 2e–h). As shown in Fig. 2f, the mean length (1.75 ± 0.13 cm) of the H_2_O_2_ treatment group became noticeably shorter relative to that of the control. Excitingly, after application with 20 mg/L IFQA, the exogenous-H_2_O_2_-induced reduction in root elongation was remarkably reversed by 29.1% (2.26 ± 0.17 cm), which was not significantly different from the control (2.46 ± 0.19 cm). The root length of the IFQA treatment group (3.17 ± 0.21 cm) was highest among the treatment groups. Meanwhile, it was observed that the inhibition of root growth on *Arabidopsis* plantlets under ATZ treatment was partially restored by IFQA (Fig. 2g). 2 µM ATZ decreased the root length to 0.86 ± 0.09 cm from the control (2.49 ± 0.09 cm). Remarkable improvement was observed in *Arabidopsis* roots (1.4 ± 0.11 cm) by the combination of ATZ and IFQA treatment (Fig. 2h). The results showed that IFQA application can promote the root elongations of maize and *Arabidopsis* under different manipulation of H_2_O_2_ levels.

### Lengths and densities of root hairs in ATZ- or H_2_O_2_-stress maize and ***Arabidopsis***

**Fig. 3:**
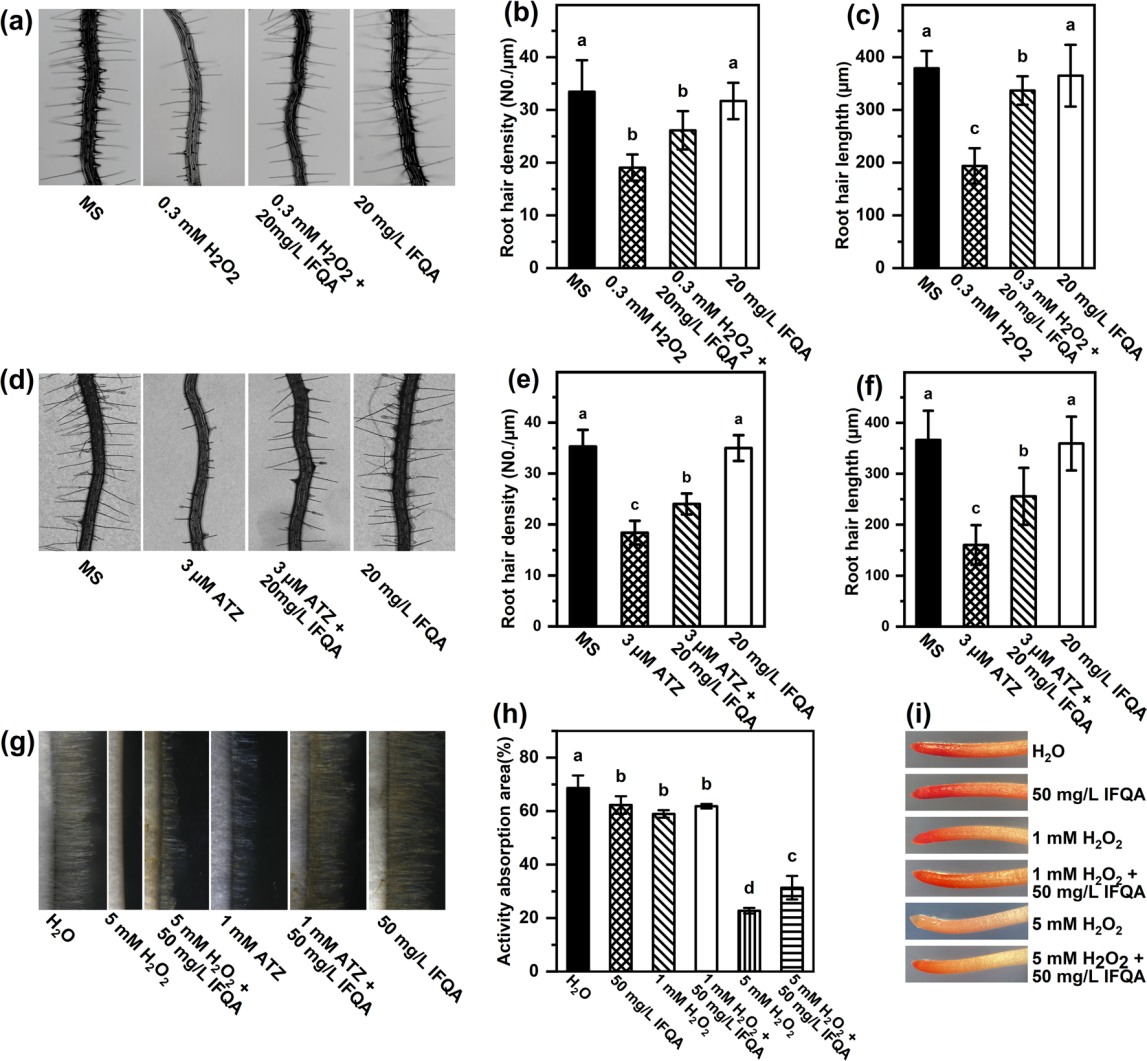
The effects of IFQA on phenotypes of root hairs, RAA, and root activity. The root-hair phenotypes (**a** and **d**), densities (**b** and **e**), and lengths (**c** and **f**) of 8-d-old *Arabidopsis* plantlets, which were planted on MS, MS + 20 mg/L IFQA, MS + 0.3 mM H_2_O_2_ + 20 mg/L IFQA, MS + 0.3 mM H_2_O_2_, MS + 3 µM ATZ, and MS + 3 µM ATZ + 20 mg/L IFQA for 8 days; The root-hair phenotypes of maize seedlings (**g**), which were cultured in water at two days after germination and then transferred into H_2_O, 50 mg/L IFQA, 5 mM H_2_O_2_, 5 mM H_2_O_2_ + 50 mg/L IFQA, 1 mM ATZ, and 1 mM ATZ + 50 mg/L IFQA for 8–12 h; The RAA (**h**) and TTC staining (**i**) of 5-d-old maize seedlings, which were cultured in water at two days after germination and then transferred into H_2_O, 50 mg/L IFQA, 1 mM H_2_O_2_, 1 mM H_2_O_2_ + 50 mg/L IFQA, 5 mM H_2_O_2_, and 5 mM H_2_O_2_ + 50 mg/L for three days. Letters above the boxes indicate statistically significant differences between samples (P < 0.05)

The root-hair zones of 7-d-old *Arabidopsis* plantlets were observed to analyze the promotional effect of IFQA on root-hair growth. As shown in Fig. 3a, the *Arabidopsis*-root hairs in the 0.3 mM H_2_O_2_ treatment group were significantly sparser than that of control. The densities of root hairs were 19.07 ± 2.49, 26.14 ± 3.63, 35.2 ± 2.15, and 31.67 ± 3.46 in the H_2_O_2_, H_2_O_2_ + IFQA, MS, and MS + IFQA treatment groups, respectively (Fig. 3b). Meanwhile, the mean length of root hairs treated by H_2_O_2_ was considerably shortest among all the treatment groups; while it was nearly restored to that of the control after addition of 20 mg/L IFQA (Fig. 3c). When 3 µM ATZ treatment on *Arabidopsis* plantlets was used to induce overaccumulation of endogenous H_2_O_2_, the root-hair formation, at the level of both density (18.36 ± 2.34) and length (160.2 ± 38.8), was inhibited (Fig. 3d–f). Under the combination of ATZ with IFQA treatment, the density (24 ± 2.05) and length (255.6 ± 55.9) of root hairs were obviously rescued, respectively (Fig. 3e, f).

The similar phenomenon for formation of root hairs has also been observed on maize seedlings. As shown in Fig. 3g, there was almost no root hair appears in the root-hair zones of maize seedlings treated by 5 mM H_2_O_2_ and the ones obviously sparser and shorter under 1 mM ATZ treatment, while it was obviously recovered after addition of IFQA. It follows that IFQA at a certain concentration can partially rescue the lengths and densities of maize- and *Arabidopsis*-root hairs under different manipulation of H_2_O_2_ levels.

### Effects of IFQA on RAA and TCC of maize roots

RAA and root activity are the most important indexes to measure root absorption function. As shown in Fig. 3h, the level of RAA was decreased to 85.9% and 33.1% in the 1 mM and 5 mM H_2_O_2_ treatment groups compared to those of the control; while, it was recovered to 90.1% and 45.8% after application IFQA, respectively. Furthermore, TTC staining was used to demonstrate the positive effect of IFQA on the root activity of maize seedlings under H_2_O_2_ treatment (Fig. 3i). The results showed that the root activity of maize roots was partially suppressed in a concentration-dependent manner by H_2_O_2_ treatment. Moreover, the inhibited root activity can be recovered after application IFQA.

### Effects of IFQA on H_2_O_2_ accumulation in maize-root tips

**Fig. 4:**
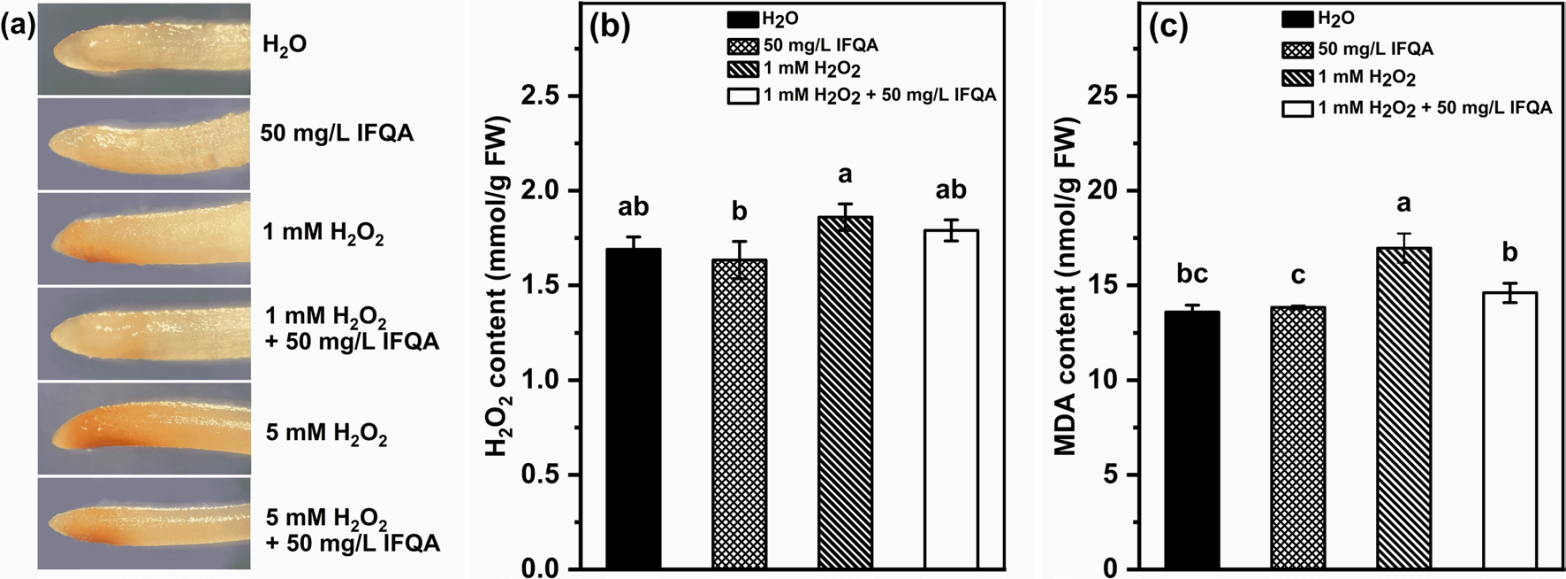
The effects of IFQA on the contents of H_2_O_2_ and MDA in maize roots under H_2_O_2_ treatment. DAB staining (**a**) of maize roots, which were cultured in water for two days after germination and then transferred into H_2_O, 50 mg/L IFQA, 1 mM H_2_O_2_, 1 mM H_2_O_2_ + 50 mg/L IFQA, 5 mM H_2_O_2_, and 5 mM H_2_O_2_ + 50 mg/L IFQA for 3 days; The contents of H_2_O_2_ (**b**) and MDA (**c**) in maize roots, which were cultured in water for two days after germination and then transferred into H_2_O, 50 mg/L IFQA, 1 mM H_2_O_2_, and 1 mM H_2_O_2_ + 50 mg/L IFQA for three days, respectively. Letters above the boxes indicate statistically significant differences between samples (P < 0.05)

It was speculated that the growth changes on roots and root hairs of maize seedlings under various treatments may be due to different levels of endogenous H_2_O_2_ accumulation. DAB staining can be used to explore the H_2_O_2_ contents in maize-root tips. As shown Fig. 4a, it was found that the staining brightness in the maize roots, especially in the root tips, was increased under H_2_O_2_ stress relative to that of the control, which means the higher level of H_2_O_2_ accumulation in maize-root tips. On the other hand, the color depth of staining in the root tips of the H_2_O_2_ + IFQA treatment group was significantly lighter than that of the H_2_O_2_ treatment group, and it was evident that the H_2_O_2_-overaccumulation level of the H_2_O_2_ treatment group was at least partially restored towards neutralization level of the control.

To investigate the regulatory role of IFQA on H_2_O_2_ accumulation in root tips, the contents of H_2_O_2_ and MDA were examined, which were commonly used to evaluate the extent of lipid peroxidation caused by oxidative stress. As shown in Fig. 4b, c, compared with the control, H_2_O_2_ treatment caused an increase of the content of H_2_O_2_ and MDA in maize roots by 10.1% and 24.2%, respectively. Upon the combination of IFQA + H_2_O_2_ treatment, the level of H_2_O_2_ accumulation and MDA content were mitigated and similar to those of the control. The findings of the comparative analysis confirmed that IFQA application alleviates the oxidative burden and restores almost completely the antioxidant pools at the lower H_2_O_2_ concentration in maize-root tips.

### IFQA regulates ASA-GSH cycle of maize roots to maintain high antioxidant potential

**Fig. 5:**
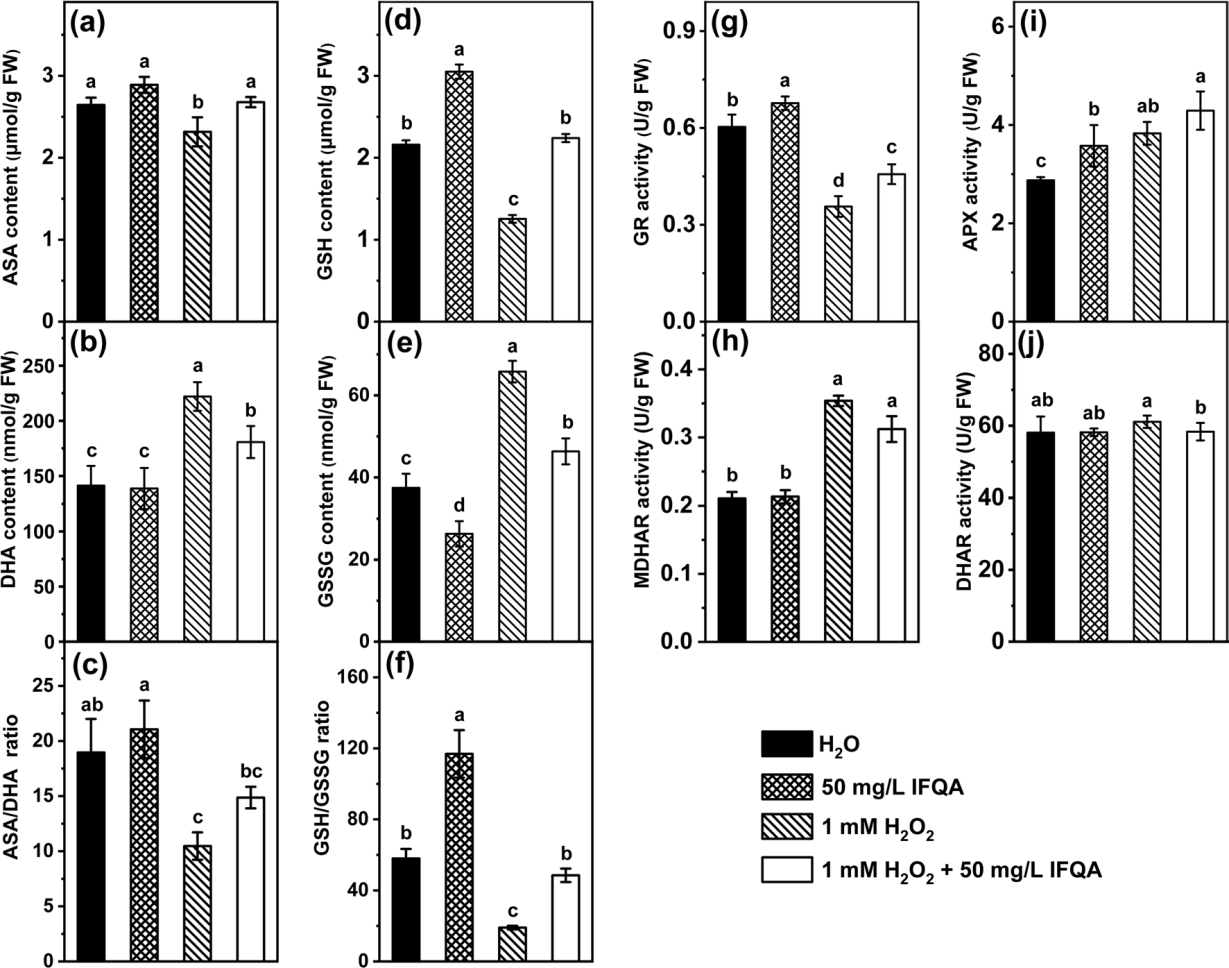
The effects of IFQA on the ASA-GSH cycle in maize roots. The contents of ASA (**a**), DHA (**b**), GSH (**d**), and GSSG (**e**), the ratios of ASA/DHA (**c**) and GSH/GSSG (**f**), and the activities of GR (**g**), MDHAR (**h**), APX (**i**), and DHAR (**j**) in maize seedlings, which were cultured in water for two days after germination and then transferred into H_2_O, 50 mg/L IFQA, 1 mM H_2_O_2_, and 1 mM H_2_O_2_ + 50 mg/L IFQA for 3 days, respectively. Letters above the boxes indicate statistically significant differences between samples (P < 0.05)

Compared with those of the control, the ASA and GSH contents of roots under H_2_O_2_ treatment exhibited a similar tendency to decrease (Fig. 5a, d); while application of the combination of H_2_O_2_ with IFQA, these contents were recovered. Especially, GSH content of the IFQA treatment group was 17.3% higher than that of the control, reaching a level around 2.4 times larger than that of the H_2_O_2_ treatment group (Fig. 5d). DHA content of maize under H_2_O_2_ treatment was significantly higher than that of the control, while the increase was reduced in the combination of H_2_O_2_ with IFQA treatment group (Fig. 5b). Similarly, the upregulated GSSG content induced by H_2_O_2_ treatment was also significantly decreased after application with IFQA; GSSG content in the IFQA treatment even displayed the lowest level (Fig. 5e). Further analysis revealed that the ASA/DHA ratio at 10.47 ± 1.24 under H_2_O_2_ treatment was the lowest level among the treatments; while the ratio (21.06 ± 2.62) under IFQA treatment was the highest level and even higher than that (18.96 ± 3.04) of the control (Fig. 5c). As shown in Fig. 5f, the variation trend of GSH/GSSG ratio was similar with the ASA/DHA ratio and even more significant. The GSH/GSSG ratio (116.90 ± 13.39) in maize under IFQA treatment is around 6 times that (19.06 ± 1.01) of H_2_O_2_ treatment; the ratio (48.50 ± 3.78) of the IFQA + H_2_O_2_ treatment was around 2.5 times that of H_2_O_2_ treatment and similar to the control.

GR, MDHAR, APX, and DHAR are also important indicators of antioxidant status of ASA-GSH cycle in plants. As shown in Fig. 5h–j, the activities of MDHAR and APX in maize roots were obviously increased under H_2_O_2_ treatment relative to that of the control; while the difference for DHAR activity was not significant. The H_2_O_2_-induced activities of MDHAR and DHAR were decreased after addition of IFQA, but the APX activity was increased. Notably, the GR activity under 1 mM H2O2 treatment was markedly decreased to 58.3% of the control, and then recovered to 76.7% of the control under the combination 1 mM H2O2 with IFQA treatment (Fig. 5g).

### IFQA Rescues reduction in Root Lengths induced by BSO

**Fig. 6:**
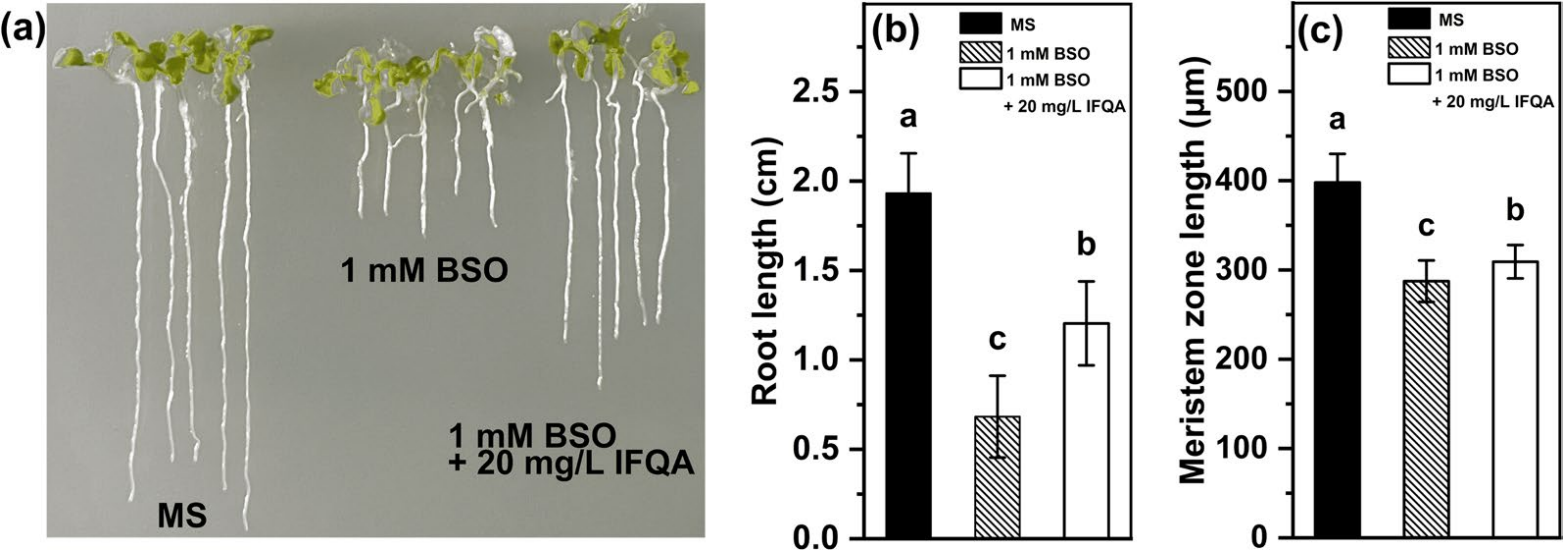
The effect of IFQA on root lengths of BSO-treated *Arabidopsis*. The phenotypes (**a**), root lengths (**b**), and meristem zone lengths (**c**) of *Arabidopsis* plantlets, which were planted on MS, MS + 1 mM BSO, and MS + 1 mM BSO + 20 mg/L IFQA for eight days. Letters above the boxes indicate statistically significant differences between samples (P < 0.05)

To determine whether IFQA-regulated GSH level is critical for root growth, *Arabidopsis* plantlets was treated by 1 mM BSO, which inhibited GSH synthesis. As shown in Fig. 6a, *Arabidopsis* root growth was reduced upon BSO treatment relative to that of the control, but the effect was undone after addition of 20 mg/L IFQA. The mean length (0.61 + 0.05 cm) of roots in the BSO treatment group was inhibited by 68.9% than that (1.96 ± 0.06 cm) of the control (Fig. 6b). The inhibited root length was partially rescued to 1.14 + 0.07 cm after application BSO combination with IFQA. The trend in the meristem zone was consistent with the root length, but the difference was not as significant as variation trend of the root lengths (Fig. 6c). It provided a direct link between application IFQA and GSH mediated-root growth.

### Effects of IFQA on NADPH-oxidase activity and H_2_O_2_ accumulation in root hairs

**Fig. 7:**
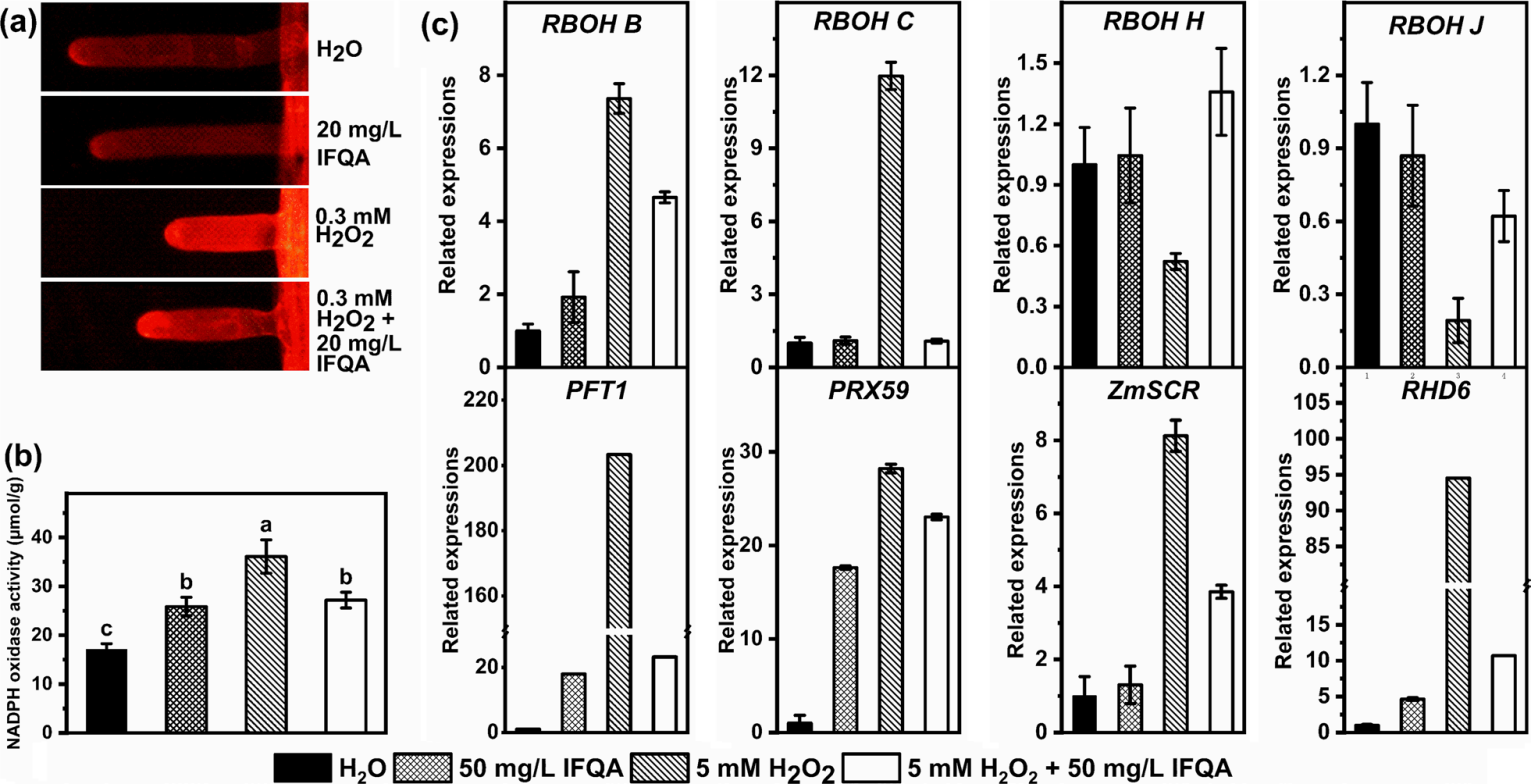
The effects of IFQA on physiological indexes and expressions of genes affecting root-hair development. The root-hair phenotypes (**a**) after ADPH probe staining for H_2_O_2_ accumulation in *Arabidopsis* plantlets, which were planted on MS, MS + 20 mg/L IFQA, MS + 0.3 mM H_2_O_2_ + 20 mg/L IFQA, and MS + 0.3 mM H_2_O_2_ for 8 days; The activities of NADPH oxidase of maize (**b**); The transcriptional expressions of genes affecting ROS producing and root-hair development (**c**) of maize seedlings, which were cultured in water for two days after germination and then transferred into H_2_O, 50 mg/L IFQA, 5 mM H_2_O_2_, and 5 mM H_2_O_2_ + 50 mg/L IFQA for three days, respectively

Due to ROS homeostasis in root-hair tips being vital for root hair development, it was reasoned that the local ROS accumulation of root hairs must present differences under the different treatments. To test the assumption, ADHP, a kind of specific H_2_O_2_ fluorescent probe, was used to explore the accumulation and distribution of H_2_O_2_ levels in root hairs. The stronger the signal intensity of fluorescence is, the higher the H_2_O_2_ level is. As shown in Fig. 7a, the fluorescent intensity in the root hairs of *Arabidopsis* plantlets was strongest and consequently provided a hint to reveal the highest ROS accumulation at root hairs under H_2_O_2_ treatment; while it was weakened under the H_2_O_2_ + IFQA treatment and lowest in the control and IFQA treatment alone. The root-hair lengths ranked by treatment groups were IFQA ≈ H_2_O > H_2_O_2_ + IFQA > H_2_O_2_, and it was shortest under H_2_O_2_ treatment (Fig. 7a). So, the results offered a direct link between root-hair growth and IFQA-regulated ROS accumulation.

NADPH oxidase can catalyze ROS production to play a vital role in regulating root-hair development; it is speculated that the changes in ROS levels of root hairs may be connected with the variation of activity of NADPH oxidase. As expected, the activity of NADPH oxidase in maize roots was increased to 2.17 times under H_2_O_2_ treatment relative to that of the control, and relieved under the combination H_2_O_2_ with IFQA treatment (Fig. 7b).

### IFQA regulates transcription of genes affecting root-hair development

To further elucidate the underlying mechanism of IFQA-regulated root-hair development, the expressions of genes affecting root-hair development of maize seedlings were performed using real-time fluorescence quantitative PCR. As shown in Fig. 7c, the class III peroxidases, *PRX59*, *RBOH B*, *RBOH C*, *RHD6*, *PFT1*, and Zm*SCR*, were demonstrated to upregulate significantly the transcript levels in maize roots of the H_2_O_2_ treatment group compared with those of the control. Inversely, the gene expressions were strongly down-regulated after addition of IFQA. However, the expression levels of *RBOH H* and *RBOH J* were decreased by H_2_O_2_ treatment, but upregulated after addition of IFQA, which was completely different with that of the above detected genes (Fig. 7c).

## Discussion

### Surface charge and aggregation properties of IFQA nanoparticles in water

In our previous report, the morphology of IFQA nanoparticles in solid was studied by scanning electron microscopy, which was non-uniform ellipsoid (long diameter: 122 nm) and further formed larger arborescent nanodendrimers [37]. For unravelling the aggregation of nanoparticles in aqueous IFQA solution at different concentration, DLS measurements were performed. The results indicated that IFQA had a monomodal and monodispersed nano-assembly tendency in water (Fig. 1 and Table 2). Similar to other amino fullerene compounds, it was supposed that the appended amino groups in IFQA, C_60_(NCH_2_CH_2_NH_3_^+^·CF_3_COO^−^)_4_·10H_2_O, were quaternization as -NCH_2_CH_2_NH_3_^+^ and ion-paired with CF_3_COO^−^ in aqueous solution [39]. 20 and 50 mg/L IFQA in deionized water were found to be positive potential by zeta potential measurement (Table 2). Furthermore, the zeta potentials were above upper limit of detection. So, IFQA in water is strongly ionized as cationic iminofullerene nanoparticles, and the aggregates can be kept separate from each other due to the electrostatic repulsion interaction between -NCH_2_CH_2_NH_3_^+^ [39]. It was interpreted that there was not a more serious aggregation trend to further form dendrimer for IFQA in water relative to that in solid. On the other hand, the positive surface charge properties of IFQA nanoparticles could allow it to be facilely absorbed by plant tissues.

### IFQA balances overaccumulational H_2_O_2_ levels through modulating ASA-GSH Cycle to enhance root growth of maize and ***Arabidopsis*** under oxidative stress

In plants, various environmental stress factors, including drought, salinity, high light, heavy metal, and high temperature, induce oxidative stress; when, upon stress, ROS production exceeds elimination, cellular homeostasis is disturbed [17, 40–45]. Therefore, ROS overaccumulation is toxic for plant and consequently leads to cell damage [18, 24, 46].

In the present study, application of IFQA can significantly alleviate the negative effects caused by different manipulation of H_2_O_2_ levels on the roots of maize seedlings and *Arabidopsis* plantlets, such as, promoting root growth and root-hair development (Fig. 2 and Fig. 3), decreasing H_2_O_2_ accumulation and MDA content (Fig. 4). It was found that a lot of the nanoparticles were absorbed onto the surface of root tips, especially root hairs (As shown in Fig. 3g, root hairs with obviously light yellow) on account of positive surface charge properties of IFQA nanoparticles (Table 2). The effect of IFQA on *Arabidopsis* plantlets is more obvious than that on maize seedlings. According to the charge properties and free radical scavenging activity in vitro of IFQA [38], it can be deduced that the absorbed IFQA onto root tips could directly act as a scavenger of free radicals removing excess ROS of outer cell layers in root tips. The more obvious effect of IFQA on *Arabidopsis* plantlets relative to that on maize seedlings may be due to less efficient penetrations of these nanoparticles to internal tissue of maize with larger roots. Obviously, the assumption of IFQA directly regulating ROS accumulation in roots needs to be further proved by more experiment evidences.

ASA-GSH cycle play key roles to maintain an appropriate ROS levels and cell redox balance in plants [29–31]. It is well established that ASA and GSH act as antioxidants associated with ROS scavenging, to regulate the critical components of the antioxidant defense system and relieve the oxidative damage caused by various stresses [28, 29, 31, 47–51].

In the present study, IFQA can increase GSH and ASA levels, reduce the amounts of GSSG and DHA, and thus improve the ratios of ASA/DHA and GSH/GSSG (Fig. 5), which reflected whether the cells were exposed to oxidative stress [52, 53]. It has been shown that the inhibited biosynthesis of GSH could repress formation of active root meristem and consequently inhibit root elongations [54]. Herein, BSO, an inhibitor of GSH synthesis, led to the inhibition of root growth, and the combination IFQA with BSO treatment partially restored the lengths of roots and meristem zones (Fig. 6). This supported the assumption that IFQA treatment was critical for root growth through regulating GSH levels to control cellular redox status of root tips (Figs. 2, 5, and 6). With the supports from the results and previous findings, it can be predicted that application of IFQA was more efficient scavenging of ROS to maintain the normal cellular redox status through the noticeable increasing in the ratios of ASA/DHA and GSH/GSSG, especially the later in maize roots under H_2_O_2_ treatment [14, 31, 53].

**Fig. 8:**
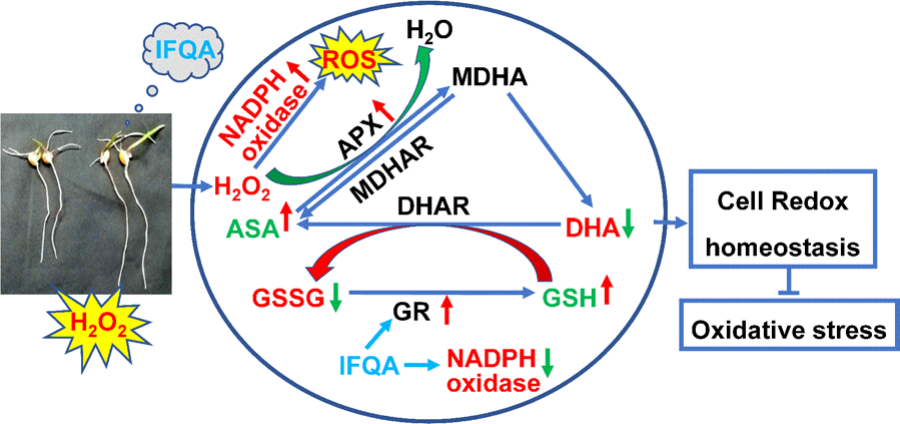
A model depicting the role of IFQA in alleviation of exogenous-H_2_O_2_-induced oxidative damage in maize roots. Short arrows beside metabolites or enzymes represent either a decrease (green) or an increase (red) in metabolite concentration or in enzyme activity, respectively

Together with the non-enzymatical antioxidants mentioned above, ROS-scavenging enzymes also participate in controlling cellular redox balance, which maintain normal ROS levels in root tips to ensure the root growth [24, 28, 55]. GR, APX, MDHAR, and DHAR are the major constitutive enzymes in ASA-GSH cycle (Fig. 8). GR can regulate the GSH/GSSG ratio through catalyzing the reduction of GSSG to GSH, which is related to the level of cell GSH library [30, 31, 56]. As shown in Fig. 4, IFQA treatment upregulated the APX activity, which can scavenge H_2_O_2_ using ASA as an electron donor and oxidize to monodehydroasorbate (MDHA, Fig. 8). Some of MDHA could be reduced to ASA by MDHAR; part of MDHA is converted to DHA. However, DHA can also be reduced to ASA with the participation of DHAR and GSH so that excess H_2_O_2_ is finally removed (Fig. 8) [31, 56]. The upregulated GR after IFQA application increased the GSH level and GSH/GSSG ratio, and consequently resulted in the decrease level of H_2_O_2_-induced oxidative stress (Fig. 5). Therefore, IFQA alleviated oxidative stress in roots through regulating the activities of related enzymes in ASA-GSH cycle to scavenge excess ROS.

## IFQA Promotes Root-hair Formation of Oxidative-stress Maize and *Arabidopsis* by Affecting NADPH-oxidase Activity and Gene Transcription

Root hairs were instrumental for plants during nutrient uptake, and its major role was to enlarge the surface area of root, thereby, facilitate absorption of water and nutrient from soil [57]. More and/or longer root hairs were beneficial to plants under drought stress or lower-nutrient conditions [58]. The absorption efficiency of the roots was depended on the number and length of the root hairs and often evaluated by the values of root activity and RAA. In the present study, the more and longer the root hairs were, the higher the root activity and RAA of maize seedlings were under IFQA + H_2_O_2_ treatment relative to that of the H_2_O_2_ treatment alone and partially explained that higher water or nutrient uptake after addition of IFQA may promote root growth (Fig. 3).

Local ROS accumulation in root-hair tips is vital for root-hair development [59]. It was also supported by our results of the experiments of the induced H_2_O_2_ accumulation, and the inhibited developments of root hairs, including length and density by exogenous H_2_O_2_ treatment, were rescued after adding IFQA (Figs. 3 and 7). This strengthens the early idea that the optimal ROS balance in root hairs for growth further imply a direct relation between root-hair growth and IFQA-regulated ROS accumulation.

Increasing evidences show that NADPH oxidase can catalyze ROS production and play an important role in regulating root-hair development [17, 25, 60]. Among of *RBOHs*, *RBOH C*, *RBOH H*, and *RBOH J* with higher ROS-producing activity are continuously required at the tips of growing root hairs [27]. Especially, *RBOH C* has been shown to control root-hair development, and the mutants without *RBOH C*/*RHD2* exhibited non-elongation of root hairs [25, 61, 62]. *RBOH H* and *RBOH J* can produce ROS that it is linked to elongation of root-hair tips at later developmental stages, which functionally overlapped with *RBOH C* [63]. In our study, 5 mM H_2_O_2_ treatment caused the significant enhancement of ROS accumulation, NADPH activity, and the expressions of *RBOH B* and *RBOH C* (Fig. 7). The moderate ROS levels was essential for root and root hair growth, however, the excess ROS accumulation inhibited the growth of roots and root hairs [10, 17, 25, 64]. It was assumed that application of IFQA decreased the expressions of *RBOH B* and *RBOH C* and the NADPH activity in the roots of maize under H_2_O_2_ treatment, and then ROS accumulation was maintained in a moderately balanceable level to result in the growth of roots and root hairs. The expression patterns of *RBOH H* and *RBOH J* in our study were different from *RBOH B* and *RBOH C* (Fig. 7), which indicated the miscellaneous regulatory pathway between of them. In addition, *PRX59*, encoding producing-H_2_O_2_ class III peroxidases, were also regulated by IFQA and predominantly expressed in the root-hair zone, supporting a possible role for root-hair formation [10, 63]. A similar trend was observed in the expression of *PFT1*, which controlled root-hair differentiation through ROS distribution in *Arabidopsis* [10]. *ROOT HAIR DEFECTIVE 6* (*RHD6*), encoding a basic Helix-Loop-Helix transcription factor, was also obviously regulated in different treatments, suggesting that *RHD6* played a crucial role in the regulation of root-hair elongation in maize [65]. Taken together, these results indicated that IFQA adjusted the formation and elongation of root hairs through balancing the ROS neutralization of root hairs and regulating the transcription of genes with ROS-mediated root-hair development.

## Conclusion

In conclusion, application of IFQA on maize seedlings and *Arabidopsis* plantlets exposed to H_2_O_2_ or ATZ treatment can maintain redox homeostasis by regulating NADPH-oxidase activity and ASA-GSH cycle to strongly scavenge free radicals, and a direct scavenging effect of IFQA may also exist. Moreover, the positive effect of fullerene-based carbon nanomaterials on maize-root-hair growth under the induced oxidative stress was discovered. IFQA can adjust root hair formation and elongation through regulating ROS neutralization of root hairs and the transcription of genes affecting ROS production and root-hair development. IFQA ameliorated oxidative stress, thereby contributing to reverse the negative effects of H_2_O_2_ accumulation on root growth and root-hair development and to increase plant resistance. The results suggested that IFQA can act as fullerene-based nanoelicitors responsible for plant growth promotion and protection from oxidative stress.

At the present stage, our studies provide a more comprehensive understanding for the promotional function and the mechanism of IFQA on plant root growth under induced oxidative stress. However, some issues require further study including cost-effective and scalable fabrication of IFQA, risk assessment to ensure a safer application. In the future, the innovative solutions of IFQA-based nanoregulator formulation and convenient application technology should be studied to alleviate oxidative stress of crop for efficient and sustainable agricultural production.

## Data Availability

Without restrictions.
